# Bewegung fördern, Gesundheit stärken

**DOI:** 10.1007/s00103-026-04321-8

**Published:** 2026-10-27

**Authors:** Susanne Krug, Kristin Manz, Anke-Christine Sass

**Affiliations:** Abteilung für Epidemiologie und Gesundheitsmonitoring, Robert Koch-Institut, Gerichtstraße 27, 13347 Berlin, Deutschland

Das Bewegungsverhalten hat sich in den vergangenen Jahrzehnten deutlich verändert. Der Wandel von körperlich aktiver Arbeit hin zu überwiegend sitzenden Tätigkeiten prägt seit Langem unseren Alltag. Gleichzeitig hat die zunehmende Nutzung des Autos unsere Alltagsmobilität und das Stadtbild verändert. Hinzu kommen neuere Entwicklungen, die Alltagsbewegung weiter reduzieren können. So ersetzen E‑Scooter mitunter kurze Fußwege, während die Digitalisierung Erledigungen, Kommunikation und Unterhaltung ohne das Zurücklegen von Wegstrecken ermöglicht. Bei Kindern und Jugendlichen kann die Nutzung von Smartphones und sozialen Medien zudem körperlich aktive Freizeitbeschäftigungen verdrängen. Seit der COVID-19-Pandemie hat auch das Arbeiten im Homeoffice deutlich an Bedeutung gewonnen, wodurch für viele Menschen Arbeitswege zumindest an einzelnen Tagen entfallen.

Gleichzeitig haben sich in den vergangenen Jahrzehnten auch positive Entwicklungen ergeben: Durch den Einsatz moderner Technik können körperlich belastende berufliche Tätigkeiten oftmals reduziert und damit arbeitsbedingte Beschwerden vermindert werden. Auch das Wissen über die gesundheitliche Bedeutung von körperlicher Aktivität ist in den vergangenen Jahrzehnten in der Bevölkerung gewachsen und der Anteil der Menschen, die in ihrer Freizeit körperlich aktiv sind und Sport treiben, ist gestiegen.

Solche gesellschaftlichen Entwicklungen verändern die Bedingungen, unter denen Menschen sich bewegen, und eröffnen neue Forschungsfragen. Vor dem Hintergrund einer wachsenden wissenschaftlichen Evidenz sowie neuer gesellschaftlicher und gesundheitspolitischer Herausforderungen werden nationale und internationale Empfehlungen für gesundheitsförderliche körperliche Aktivität daher regelmäßig überprüft und weiterentwickelt.

Aktuelle Daten zum Bewegungsverhalten zeigen, dass sowohl bei Kindern und Jugendlichen als auch bei Erwachsenen ein Großteil der Bevölkerung zu inaktiv ist und nationale und internationale Bewegungsempfehlungen nicht erfüllt. Die Weltgesundheitsorganisation (WHO) empfiehlt Kindern und Jugendlichen im Durchschnitt mindestens 60 min körperliche Aktivität pro Tag, ergänzt durch intensive und muskelkräftigende Aktivitäten an mehreren Tagen pro Woche. Erwachsene sollten sich mindestens 150 min pro Woche mit moderater Intensität bewegen und an mindestens 2 Tagen pro Woche muskelkräftigende Aktivitäten einbauen. Von körperlicher Inaktivität sind insbesondere Personen mit niedrigem sozioökonomischen Status betroffen. Bewegungsmangel ist daher zu einer zentralen Herausforderung für Public Health geworden.

Je stärker veränderte Arbeitsbedingungen und Digitalisierung körperliche Aktivität im Alltag überflüssig machen, desto wichtiger wird es, Bewegung in allen Bevölkerungsgruppen bewusst zu ermöglichen, zu fördern und in alltägliche Lebenswelten zu integrieren. Denn regelmäßige körperliche Aktivität trägt wesentlich zur Prävention zahlreicher Erkrankungen sowie zur Entwicklung und zum Erhalt von Mobilität, Selbstständigkeit, Leistungsfähigkeit und Lebensqualität über den gesamten Lebensverlauf bei. Eine internationale Studie schätzt den Anteil der Todesfälle in Deutschland, der auf körperliche Inaktivität zurückzuführen ist, auf rund 7,5 % [1]. Zudem werden die allein durch körperliche Inaktivität verursachten direkten Gesundheitskosten für Deutschland auf rund 2 Mrd. € jährlich geschätzt [2]. Das Gesundheitssystem steht durch den demografischen Wandel, den zunehmenden Fachkräftebedarf in der Pflege, steigende Gesundheitsausgaben sowie wachsende Anforderungen infolge chronischer Erkrankungen vor grundlegenden Herausforderungen. Damit wird es wichtiger, gesundheitspolitisch nicht erst bei der Behandlung von Erkrankungen anzusetzen, sondern Prävention und Gesundheitsförderung – einschließlich der Bewegungsförderung – systematisch zu stärken. Eine Steigerung der körperlichen Aktivität kann maßgeblich zur Prävention zahlreicher nichtübertragbarer Erkrankungen beitragen, die Lebensqualität der Menschen erhöhen und zugleich das Gesundheitssystem entlasten.

Ziel der Bewegungsförderung sollte es sein, Bewegung nicht allein der persönlichen Motivation und Organisation zu überlassen, sondern sie als selbstverständliche, zugängliche und gesundheitsförderliche Option im Alltag zu verankern. Dazu sind Verhältnisse erforderlich, die aktive Mobilität, Spiel, Sport und Alltagsbewegung erleichtern: sichere Fuß- und Radwege, bewegungsfreundliche Kitas und Schulen, attraktive öffentliche Räume und Flächen in jedem sozialen Umfeld, bewegungsförderliche Arbeitsbedingungen sowie niedrigschwellige und bezahlbare Sport- und Freizeitangebote. Ebenso bedarf es einer politikfeld- und sektorübergreifenden Zusammenarbeit zur Stärkung der Bewegungsförderung, die in Deutschland im Jahr 2022 mit dem Runden Tisch „Bewegung und Gesundheit“ unter Leitung des Bundesministeriums für Gesundheit vielversprechend begonnen hat.

Gleichzeitig ergeben sich vielfältige Möglichkeiten zur großflächigen Bewegungsförderung, die nicht ungenutzt bleiben sollten. Zu nennen sind beispielsweise die notwendigen Umbrüche im Mobilitätssektor und im Städtebau aufgrund der Herausforderungen der Klimakrise. Diese urbane Transformation bietet die Chance, Lebensräume bewegungsfreundlich zu gestalten, wovon alle Bevölkerungsgruppen profitieren können. Auch das zunehmende Angebot der Ganztagsbetreuung in Schulen bietet die Aussicht, mehr Kindern einen niedrigschwelligen Zugang zu Bewegungsangeboten zu ermöglichen. Chancen bietet zudem die Digitalisierung: Digitale Medien und soziale Netzwerke können dazu beitragen, niedrigschwellig zu Bewegung zu motivieren, Menschen miteinander zu vernetzen und gemeinsame körperliche Aktivitäten auch im realen Lebensraum anzustoßen. Dies kann insbesondere für Personen hilfreich sein, denen der Zugang zu bestehenden Bewegungs- und Sportangeboten bislang erschwert ist. Um gesundheitsbezogener Ungleichheit entgegenzuwirken, ist es wichtig, die Voraussetzungen und Bedarfe von benachteiligten Gruppen zu berücksichtigen, insbesondere von Personen mit niedrigem sozioökonomischen Status und von Menschen mit Behinderungen.

Das letzte Themenheft zu „Körperlicher Aktivität“ erschien im Januar 2012 [3]*.* Im Mittelpunkt standen die gesundheitlichen Wirkungen und die Messung körperlicher Aktivität sowie die Verbreitung von Bewegung in der Bevölkerung. Daran anknüpfend geht das vorliegende Heft „Bewegung und Bewegungsförderung“ der zentralen Frage nach, wie körperliche Aktivität unter unterschiedlichen gesellschaftlichen und individuellen Voraussetzungen nachhaltig gefördert werden kann. Es gibt einen Überblick über Ansätze der Bewegungsförderung und beleuchtet unterschiedliche Zielgruppen und Lebenswelten, strukturelle Voraussetzungen, die praktische Umsetzung sowie zukünftige Herausforderungen wie Digitalisierung, Klimawandel und Monitoring.

Das Heft wird durch 2 Übersichtsartikel mit hoher Public-Health-Relevanz eröffnet. Sie greifen die beiden Themen auf, bei denen körperliche Aktivität eine wichtige Rolle in der Gesundheitsförderung spielt: die Prävention von Adipositas (*Joisten et al.*) und die Förderung der psychischen Gesundheit (*Reiß et al.*).

Danach folgen 5 Schwerpunktartikel mit Praxisbezug, die Ansätze der Bewegungsförderung in unterschiedlichen Lebenslagen vorstellen – von Menschen mit Beeinträchtigung (*Eghbalpour et al.*) und Menschen mit Migrationsgeschichte (*Abu-Omar et al.*) über Kinder und Jugendliche in der Ganztagsschule (*Neuber et al.*) und der offenen Kinder- und Jugendarbeit (*Voigts et al.*) bis hin zu älteren Menschen (*Aluttis et al.*). Es wird deutlich, dass nachhaltige Bewegungsförderung zielgruppenspezifisch gestaltet und an den jeweiligen Lebenswelten ausgerichtet sein muss. Sie erfordert geeignete Strukturen, qualifizierte Akteurinnen und Akteure sowie eine gute Zusammenarbeit zwischen Sport, Bildung, sozialer Arbeit und kommunaler Politik.

Drei weitere Beiträge richten den Blick auf zukünftige Herausforderungen und Potenziale der Bewegungsförderung. Sie zeigen, wie digitale Technologien eine personalisierte Unterstützung ermöglichen *(Kunst et al.*), welche strukturellen und individuellen Barrieren beim alltäglichen Fahrradfahren zu berücksichtigen sind (*Manz et al.*) und wie der Klimawandel insbesondere durch Hitze das Bewegungsverhalten beeinflussen kann (S. *Schneider und S. Mencocco*). Gemeinsam verdeutlichen sie die Notwendigkeit innovativer, zielgruppenspezifischer und an veränderte gesellschaftliche sowie ökologische Bedingungen angepasster Ansätze.

Qualitativ hochwertige Daten bilden die Grundlage für eine zielgerichtete Bewegungsförderung. Sie geben Aufschluss über das Bewegungsverhalten der Bevölkerung, seine zeitliche Entwicklung sowie zielgruppenspezifische Bedingungen, die gesundheitsförderliches Bewegungsverhalten erleichtern oder erschweren. Den Abschluss des Heftes bildet daher ein methodischer Artikel, der Unterschiede zwischen globalen und nationalen Prävalenzschätzungen zu körperlicher Aktivität einordnet und methodische Fallstricke aufzeigt, die es dabei zu lösen gilt (*Kossmann et al.*).

Wir hoffen, dass die Beiträge dieses Schwerpunkthefts neue Perspektiven auf die Bewegungsförderung eröffnen und zum Weiterdenken anregen – vielleicht auch bei einem anschließenden Spaziergang.

## Data Availability

Für diesen Artikel wurden keine Datensätze erstellt oder analysiert.
